# Neutrophil to Lymphocyte and Lymphocyte to Monocyte Ratios Predict Improved Survival and Response to Induction Chemotherapy in Locally Advanced Squamous Cell Carcinoma of the Larynx

**DOI:** 10.1002/hed.70132

**Published:** 2025-12-16

**Authors:** Zachary Risch, Emily Bellile, Moneb S. Bughrara, Paul L. Swiecicki, Keith Casper, Kelly Malloy, Norman Hogikyan, Matthew Spector, Andrew Shuman, Chaz Stucken, Steven Chinn, Douglas C. Chepeha, Shruti Jolly, Michelle Mierzwa, Carol Bradford, Avraham Eisbruch, Thomas Carey, Mark Prince, Gregory T. Wolf, Francis P. Worden

**Affiliations:** ^1^ Department of Internal Medicine, Division of Hematology/Oncology Ohio State University Columbus Ohio USA; ^2^ Cancer Data Science, Department of Biostatistics University of Michigan School of Public Health Ann Arbor Michigan USA; ^3^ Department of Internal Medicine University of Michigan Ann Arbor Michigan USA; ^4^ Department of Internal Medicine, Division of Hematology/Oncology University of Michigan Ann Arbor Michigan USA; ^5^ Department of Otolaryngology‐Head and Neck Surgery University of Michigan Ann Arbor Michigan USA; ^6^ Department of Otolaryngology‐Head and Neck Surgery University of Pittsburgh Pittsburgh Pennsylvania USA; ^7^ Department of Radiation Oncology University of Michigan Ann Arbor Michigan USA; ^8^ Department of Otolaryngology‐Head and Neck Surgery Ohio State University Columbus Ohio USA

**Keywords:** induction chemotherapy, lymphocyte/monocyte, neutrophil/lymphocyte ratio

## Abstract

**Background:**

University of Michigan Cancer Center (UMCC) protocol 9520 treated stage III/IV locally advanced squamous cell carcinoma of the larynx (LASCCL) with cisplatin and 5‐fluorouracil to select for definitive therapy based on response. Studies have shown that neutrophil‐lymphocyte ratio (NLR) and lymphocyte‐monocyte ratio (LMR) are potential prognostic markers in p16‐negative LASCCL. This study analyzes the predictive value of NLR and LMR.

**Methods:**

Samples from 193 LASCCL patients treated with chemotherapy were reviewed. Response to induction chemotherapy was tested with logistic regression. Optimal cut‐points were determined by Youden's index. Survival was tested with Cox proportional hazards models.

**Results:**

LMR had a positive association, NLR had a negative association with response to chemotherapy (*p* = 0.004;0.07). Response was higher in patients with LMR ≥ 2.8 (*p* = 0.0007) and NLR ≤ 2.8 (*p* = 0.04). Overall and disease‐specific survival improved with LMR ≥ 2.8 (*p* = 0.0002;0.004) and NLR ≤ 2.8 (*p* = 0.10;0.03).

**Conclusions:**

Low NLR and high LMR were associated with favorable responses to chemotherapy and survival in LASCCL.

## 1 Introduction

There are approximately 13 020 new cases of laryngeal cancer (LASCCL) in the United States resulting in 3910 deaths annually [1]. Prior to the early 1990s, standard treatment for LASCCL was total laryngectomy and adjuvant radiation therapy (RT), culminating in 5‐year survival rates of approximately 50% with high rates of functional morbidity [2, 3, 4, 5, 6]. In 1991, the Department of Veteran's Affairs (VA) laryngeal cancer group study demonstrated high rates of laryngeal preservation using induction chemotherapy followed by radiation [7]. RTOG 91–11 established concurrent chemoradiation as standard of care for larynx preservation for LASCCL [8].

Patients not responding to chemoradiation (CRT) or RT are subject to salvage surgery with the increased risk for surgical complications [9]. Thus, identifying patients unlikely to respond to CRT may improve survival and reduce acute and long‐term complications. The University of Michigan conducted a phase II clinical trial (UMCC 9520) with the hypothesis that one cycle of induction chemotherapy (IC) would select patients for laryngeal preservation versus laryngectomy upfront. This treatment strategy demonstrated high rates of laryngeal preservation and overall survival as well as improvements in voice‐ and swallowing‐related quality of life [10, 11]. Confirmatory data from our institution (UMCC 9520‐like) confirmed that exceptional survival rates were again achieved with a bioselective treatment approach utilizing one cycle of neoadjuvant chemotherapy [12].

Correlative data from the UMCC 9520 study revealed high levels of peripheral CD4+ lymphocytes predicted response to induction chemotherapy and improved survival, suggesting that a biomarker could be useful for predicting who might benefit from chemoradiation without bioselection from IC [13]. Based on these correlative findings, our present analysis was designed to test the utility of pre‐treatment NLR and LMR as biomarkers for response to induction chemotherapy. Neutrophil to lymphocyte ratios (NLR) and lymphocyte to monocyte ratios (LMR) are prognostic for various solid tumors, including squamous cell carcinoma of the head and neck (SCCHN) [14, 15, 16]. Unlike all other NLR/LMR biomarker studies, our study tested these biomarker ratios from prospectively collected specimens under an IRB approved clinical trial for laryngeal preservation.

## 2 Methods

### 2.1 Study Design and Data Sources

**FIGURE 1:**
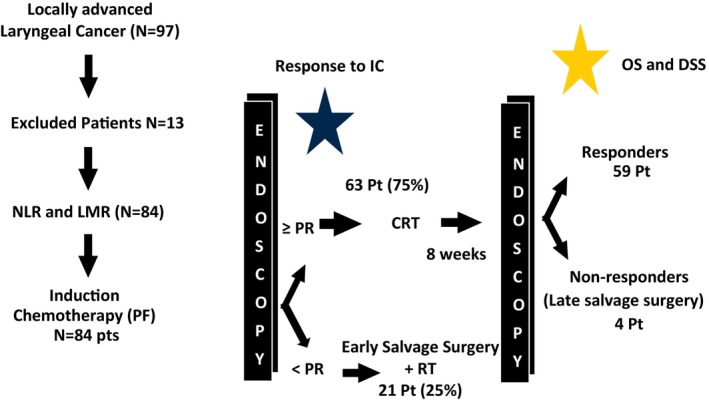
Schematic of study design. UMCC 9520 was a single institution protocol in which patients with stage III/IV locally advanced LSCC were treated with 1 cycle of PF. NLR & LMR were calculated from pre‐treatment CBC. Patients whose tumors attained a < 50% response, measured by direct laryngoscopy, were treated with total laryngectomy + RT. Those who had a > 50% response to IC underwent CRT. Repeat direct laryngoscopy was done 8 weeks after completion of CRT. Patients who showed disease persistence or recurrence underwent salvage total laryngectomy. Outcomes measured were response to IC (blue star) and OS and DSS (maize star). [Color figure can be viewed at wileyonlinelibrary.com]

Data was obtained from two primary data sources for this study. The first was from a prospective phase II clinical trial (UMCC 9520) conducted at the University of Michigan from 1995–2000. In a confirmatory dataset, patients were subsequently treated with the same regimen off study (aka UMCC 9520‐like) from 2002–2018. Patients from the UMCC 9520 and UMCC 9520‐like cohorts had locally advanced laryngeal cancer (stage III/IV) and were candidates for total laryngectomy. Patient characteristics are noted in Table 1. All patients were staged with direct laryngectomy (DL) and contrast‐enhanced computed tomography (CT). All patients were given 1 cycle of cisplatin 100 mg/m^2^ on day 1 and fluorouracil 1000 mg/m^2^/day by continuous infusion over 5 days. All eligible patients underwent DL to assess tumor response 3 weeks post IC. Response was classified as CR: 100% resolution, PR: ≥ 50% dimensional reduction, or non‐responders: < 50% dimensional reduction. All responders (CR, PR) received definitive CRT, consisting of daily fractionated RT to 70 Gy in 2 Gy per fraction with concurrent cisplatin 100 mg/m^2^ every 3 weeks. Patients who were deemed non‐responders (NR) underwent total laryngectomy followed by adjuvant RT. Patients with relapse or recurrence after CRT underwent salvage laryngectomy (UMCC 9520 Study Schema Figure 1).

**TABLE 1:** Clinical and demographic characteristics all subjects, *n* = 193.

Variable	Mean (std) or *n* (%)
Age at Dx	
Years	58.3 (10.0)
Sex	
M	149 (77%)
F	44 (23%)
Main tumor site	
Glottic	48 (25%)
Supraglottic	138 (72%)
Pyriform Sinus	7 (4%)
Clinical stage	
III	71 (37%)
IV	122 (63%)
Tstage	
1, 2	15 (8%)
3	104 (54%)
4	74 (38%)
Smoking	
Never	6 (3%)
Past	50 (26%)
Current	135 (70%)
Missing data	
Response to IC	
≥ 50%	150 (78%)
< 50%	43 (22%)

Data was collected from the electronic medical record. NLR and LMR were calculated from pretreatment CBCs for all patients. Patients were excluded from the analysis for incomplete or missing CBC data, use of concurrent corticosteroids, documentation of active infections, known histories of chronic lymphocytic leukemia, or other medical conditions known to alter neutrophils or lymphocytes.

### 2.2 Statistical Analysis

Associations between ratios and the probability of response to IC were tested with logistic regression. We estimated that a two‐sided test of proportion between biomarker high and low groups was powered (> 80%) to detect a minimum difference in IC response of > 40% for NLR and > 30% for LMR. Power was evaluated assuming an allowable type I error of 0.05 and the data we had available categorized by historical cut points obtained from the literature. For analysis, ratios were log‐transformed and treated as continuous in exploratory models. Binary cut points were defined as optimized values for predicting the probability of IC response based on Youden's Index in the current data [15, 16].

The odds ratio (OR), sensitivity, specificity, positive predictive value (PPV), and negative predictive value (NPV) for response to IC were calculated using standard methods. Overall and disease‐specific survival (OS and DSS, respectively) were defined from the date of diagnosis to the date of death or last follow‐up. Death from causes other than laryngeal cancer was censored at the date of death for DSS. Cox proportional hazards models were used for testing associations with survival. The proportional hazards assumption was tested using time‐dependent covariates in each model. Spearman correlation coefficients were calculated to estimate associations between the ratios (NLR, LMR) and peripheral blood lymphocyte counts (CD4, CD8).

## 3 Results

### 3.1 Study Population

UMCC 9520 consisted of 97 patients with locally advanced laryngeal cancer. Of the 97 patients, two were lost to follow‐up. Of the remaining patients, eight did not have CBC data available for review in the dataset or medical record, and three were excluded due to corticosteroid use or a documented infection at the time of the pretreatment CBC collection. The final sample comprised data from 84 patients.

There were 126 patients in the UMCC 9520‐like data set. Of the 126 patients, 17 were excluded (seven concurrent steroid use, six missing CBC data, three concurrent infection, and one with CLL), resulting in 109 patients. The combined study population consisted of 193 patients.

### 3.2 Response to Induction Chemotherapy

Optimal cut points (2.8 for both NLR/LMR) for predicting response to IC based on Youdin's index were determined. Results of logistic regression predicting response to induction chemotherapy, sensitivity, specificity, PPV, and NPV are summarized in Table 2. Ninety‐nine of 193 patients had NLR ≤ 2.8 and 102 of 193 had LMR ≥ 2.8. Of the 99 patients with an NLR ≤ 2.8, 83 were responders with a PPV of 84%, sensitivity of 0.55, and specificity of 0.63. Of the 102 patients with an LMR ≥ 2.8, 87 were responders with a PPV of 85%, sensitivity of 0.58, and specificity of 0.65. Subset analyses by T stage and subsite were performed and summarized in Table S1.

**TABLE 2:** Outcome measures of response to IC based on ratio.

	Marker positive	IC responders among marker positive	Sensitivity	Specificity	PPV	NPV	Odds ratio (95% CI)	*p*
NLR ≤ 2.8	99/193	83/99	0.55	0.63	84%	29%	2.09 (1.04, 4.20)	0.04
LMR ≥ 2.8	102/193	87/102	0.58	0.65	85%	31%	2.58 (1.27, 5.22)	0.0007
NLR ≤ 2.8 and LMR ≥ 2.8	79/193	68/79	0.45	0.74	86%	28%	2.41 (1.13, 5.14)	0.02

*Note*: NLR and LMR cutoff values of 2.8 based on cut‐point analysis optimizing Youdin's index. Odds ratio from logistic regression for P (response to IC), sensitivity, specificity, PPV, and NPV for response to IC were calculated.

### 3.3 Overall and Disease Specific Survival

**FIGURE 2:**
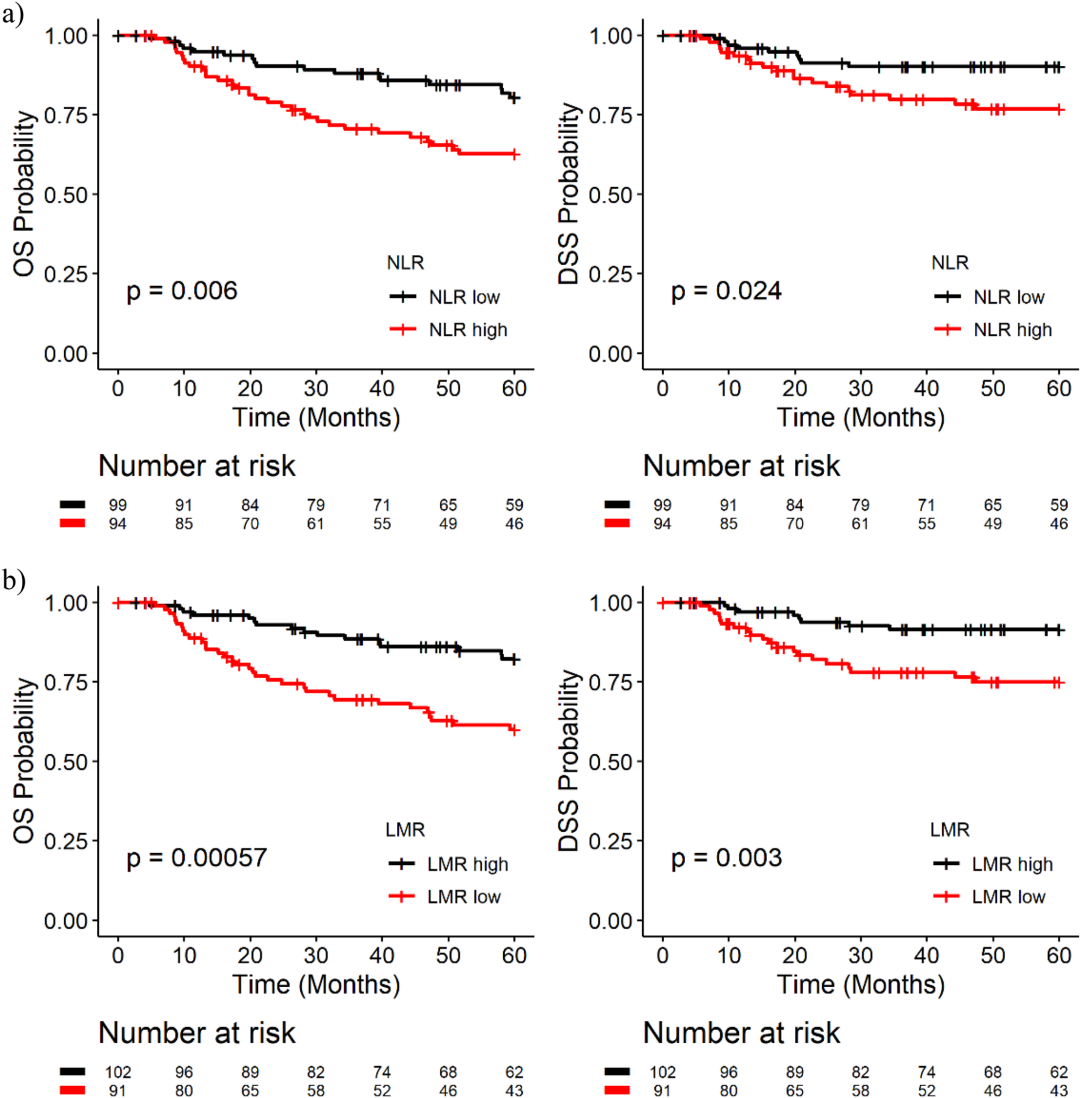
Ratios and survival over time. Kaplan Meier curves of overall, disease‐specific survival time stratified by 2.8 cut‐point based on (a) NLR (b) LMR. [Color figure can be viewed at wileyonlinelibrary.com]

Lower NLR and higher LMR ratios were each associated with better overall and disease‐specific survival. The prognostic significance of NLR or LMR in OS and DSS is reported in Table 3. Results are shown for the whole cohort and stratified by the T stage. When classifying “good” and “poor” ratios with the proposed 2.8 cut point, low NLR was associated with a 40%–50% reduction in hazard with a hazard ratio (95% CI) of 0.6 (0.4, 1.0) and 0.5 (0.2, 0.9) for OS and DSS, respectively. High LMR was associated with a 60% reduction in hazard; hazard ratio (95% CI) of 0.4 (0.2, 0.6) and 0.4 (0.2, 0.8) for OS and DSS, respectively. Survival curves are displayed, stratified by the cut points (Figure 2). The proportional hazards assumption was tested and no violations were detected via time‐dependent covariates.

**TABLE 3:** Overall (OS) and disease‐specific survival (DSS) based on ratios and stratified by Tstage.

Quantification	Ratio	Dataset	OS			DSS		
			HR	95% CI	*p*	HR	95% CI	*p*
Continuous	Log NLR	All	**3.6**	**1.4–9.0**	**0.008**	**4.1**	**1.1–14.6**	**0.03**
		T4 only	2.6	0.6–10.6	0.19	4.5	0.7–29.1	0.11
	Log LMR	All	**0.1**	**0.05–0.3**	**< 0.0001**	**0.2**	**0.04–0.7**	**0.01**
		T4 only	**0.1**	**0.02–0.5**	**0.005**	**0.1**	**0.01–1.0**	**0.05**
Cut points	NLR **≤** 2.8	All	**0.6**	**0.4–0.9**	**0.02**	0.6	0.3–1.0	0.06
		T4 only	0.8	0.4–1.6	0.47	0.6	0.2–1.6	0.33
	LMR ≥ 2.8	All	**0.4**	**0.2–0.6**	**< 0.0001**	**0.4**	**0.2–0.8**	**0.009**
		T4 only	**0.3**	**0.1–0.7**	**0.003**	**0.3**	**0.1–0.9**	**0.03**
Combination	NLR good, LMR good	All	Ref			Ref		
	NLR poor, LMR good		0.9	0.4–2.2	0.80	1.0	0.3–3.2	0.96
	NLR good, LMR poor		**2.5**	**1.2–4.6**	**0.02**	1.7	0.5–5.4	0.35
	NLR poor, LMR poor		**2.7**	**1.6–4.6**	**0.002**	**2.5**	**1.2–5.1**	**0.01**

*Note*: The bolded values represented statistical significance.

Abbreviation: HR, hazard ratios (HR) from unadjusted Cox proportional‐hazard model with a single covariate.

**FIGURE 3:**
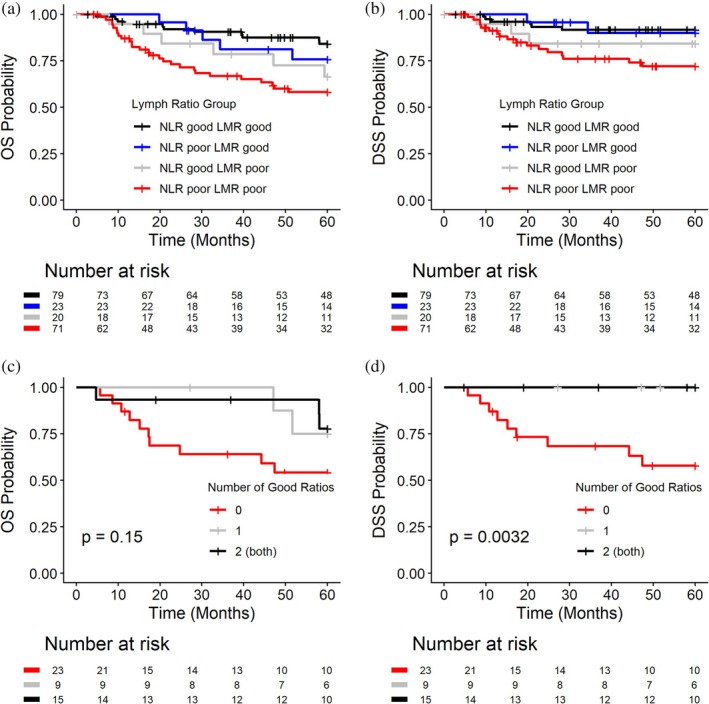
(a and b) Ratios and survival over time. Kaplan Meier curves of overall, disease‐specific survival time stratified by combination of NLR and LMR cut‐points. *p*‐values for pairwise comparisons available in Table 3. (c and d) T4 Supraglottic subset analysis. Kaplan Meier curves overall, disease‐specific survival time stratified by risk score based on NLR and LMR in T4 supraglottic subset. [Color figure can be viewed at wileyonlinelibrary.com]

Survival curves, when categorizing patients into risk groups based on combinations of NLR and LMR in terms of good and poor ratios, are displayed in Figure 3a,b; hazard ratios are reported in Table 3 in the group as a whole and stratified by T stage. The group with the poorest survival had “poor” NLR and “poor” LMR ratios followed by those with good NLR but poor LMR ratios, demonstrating the importance of lymphocyte counts in relation to neutrophils and monocytes. A subset analysis among T4 supraglottic patients was performed, and the survival advantage of good LMR and NLR is displayed in Figure 3c,d.

To evaluate whether absolute lymphocyte counts are the main driver in the survival associations, we performed Cox models for NLR and LMR and their combinations, adding absolute lymphocyte counts (ALC) as a fixed covariate in each model (Table S2). After controlling for ALC, the significance of associations with survival remains for LMR and the combination group.

### 3.4 Laryngectomy Free Survival Among Patients Treated With CRT

In a subset analysis, among patients who underwent definitive CRT, updated NLR and LMR ratios were obtained from immediately preceding CRT and analyzed for an association with Laryngectomy Free Survival. When classifying “good” and “poor” ratios with the proposed 2.8 cut point, low NLR was associated with a 60% reduction in hazard with a hazard ratio (95% CI) of 0.4 (0.2, 1.0). High LMR was not significantly associated with a reduction in hazard; the hazard ratio (95% CI) was 0.6 (0.2, 1.5).

### 3.5 Association Between Ratios and Peripheral Blood Lymphocytes

Figure S2 summarizes the association between NLR and LMR and peripheral blood lymphocytes from previously published correlative data from UMCC 9520 [13]. As expected, CD4 and CD8 counts were positively correlated with LMR and inversely correlated with NLR ratios. Among those with T4 tumors, the strength of the associations was not as robust, which may be due to small sample size or perhaps the aggressive nature of this tumor population.

## 4 Discussion

Standard of care for patients with locally advanced laryngeal cancer, in particular T4 tumors, is total laryngectomy. However, cisplatin and radiation therapy have become standard for organ preservation. Current guidelines suggest that swallowing and voice quality of life are a benefit of chemoradiation [11]. We previously demonstrated that bioselection with a single cycle of IC can effectively select patients for chemoradiotherapy [12]. Valid biomarkers to predict IC response have yet to be established. In our analysis, lower NLR and higher LMR are associated with response to IC and better OS and DSS. The positive predictive value (PPV) for predicting response to IC with the combination of NLR and LMR was 86%. Our study suggests that nearly 50% of patients who received IC for bioselection could have foregone IC based on NLR/LMR derived from a pretreatment blood sample.

We examined prospectively collected biomarkers from an organ preservation trial (UMCC 9520) and patients treated subsequently using the same bioselection methodology. IC response was determined via direct visualization by the treating surgeon. We are aware, nonetheless, that this method has the potential to introduce bias due to the variability of those assessing response. Moreover, as we expanded this approach outside of the clinical trial, more inter‐provider variability was possible. We noted, however, that the associations between lymphocyte ratios from peripheral blood, response to IC, and survival (OS, DSS) remained consistent within the trial and real‐world cohort. This suggests biomarkers may eliminate clinical bias to select patients for organ preservation.

Various cut points for NLR and LMR can be found in the literature. One meta‐analysis demonstrated significant differences in OS for NLR cutoff values < 2 and ≥ 6, but no significant difference in OS for ratios between 2–6 [16]. Another study of non‐HPV oropharyngeal cancer used NLR of 4.7 and found prognostic significance [14]. A 2015 meta‐analysis demonstrated that LMR < 3 was associated with the largest magnitude of effect on OS in a variety of solid tumors [17]. Given that these studies were performed for a variety of solid tumors with different treatment paradigms, we performed our own internal analysis to define cut point values to predict response to IC in laryngeal squamous cell cancer patients. Furthermore, we controlled our analysis by excluding patients whose lymphocytes/neutrophils may have been influenced by underlying medical conditions or medications (i.e., infection, use of systemic steroids).

Utilizing our internally derived cut points, we examined the association between pre‐CRT NLR and LMR and laryngectomy‐free survival. Patients with a low NLR experienced a 60% reduction in the hazard of laryngectomy‐free survival compared with those with a high NLR, whereas LMR was not significantly associated with the outcome. Gorphe et al. conducted an observational cohort study of patients receiving induction chemotherapy for larynx preservation and demonstrated that an NLR > 5 predicted poor outcomes independent of response to induction chemotherapy [18]. Similarly, Zeng et al. reported an observational study of patients treated with definitive chemoradiotherapy and found that an NLR < 3 was associated with significantly better rates of overall survival, disease‐free survival, and locoregional control [19]. However, the association of LMR with laryngectomy‐free survival is less established in the literature.

Within UMCC 9520, a significant positive correlation with LMR and a significant inverse correlation with NLR was noted for CD4+ lymphocyte counts within peripheral blood (Figure S2). Immunoscoring based on quantification of CD3+ and CD8+ TILs is thought to be clinically prognostic [20]. Elevated CD4+ and CD8+ tumor infiltrating lymphocytes (TILs) are associated with both improved disease‐free survival and DSS in persistent/recurrent laryngeal squamous cell carcinoma [21]. Additionally, elevated CD3+ and CD8+ TILs are associated with improved OS after definitive CRT in HPV‐negative head and neck SCC [22]. TILs could thus serve as tissue biomarkers for selecting patients who may benefit from CRT. Likewise, peripheral blood LMR significantly correlates with CD4+ and CD8+ TILs in localized esophageal squamous cell carcinoma [23]. It is unclear if peripheral blood lymphocyte counts correlate with tumor infiltrating lymphocyte counts (TILs) within the tissue of laryngeal squamous cell carcinoma.

Ultimately, our goal is to biologically select patients for chemoradiotherapy in lieu of induction chemotherapy utilizing a combination of objective markers. Fifty‐three percent of patients in our analysis had favorable LMR. This suggests that 53% of future patients would be eligible for chemoradiation upfront with an LMR ≥ 2.8, eliminating the need for bioselection with IC. On the contrary, the low sensitivity (0.45) and low negative predictive value (28%) from our data suggest patients with LMR < 2.8 should receive IC for bioselection. Patients with LMR < 2.8 (poor) but NLR ≤ 2.8 (good) had slightly improved survival compared to those with NLR > 2.8 (poor) and LMR < 2.8 (Figure 3); however, the size of this group is too small for us to conclude that bioselection is not warranted.

In summary, we evaluated the utility of NLR and LMR as biomarkers for response to IC as well as survival in patients with locally advanced laryngeal cancer treated under an organ preservation paradigm. Our data is the first to demonstrate NLR and LMR are associated with response to IC and increased OS and DSS. Additionally, low NLR prior to CRT was associated with increased rates of LFS. This study is limited by its retrospective nature. However, our data was compiled from prospectively collected blood samples from our nationally registered clinical trials and patients were treated with the exact same bioselection strategy. Furthermore, due to the limitations of the size of our population, several confidence intervals, although significant, remained wide. While we recognize the potential for bias introduced from evaluations performed by different surgeons, the associations of NLR and LMR with IC response and overall and disease‐specific survival remained consistent throughout, further substantiating the utility of NLR and LMR as clinical biomarkers.

## Funding

This work was supported by the National Cancer Institute of the National Institutes of Health under the University of Michigan Comprehensive Cancer Center Core Grant (P30 CA046592).

## Conflicts of Interest

Zachary Risch: Stock and Other Ownership Interests—Alnylam; Asterias Biotherapeutics; Geron; Oncolytics; Pfizer; Sangamo Bioscience. Paul Swiecicki: Research Funding—Ascentage Pharma Group; Pfizer. Shruti Jolly: Honoraria—Varian Medical Systems; Consulting or Advisory Role—AstraZeneca; Varian Medical Systems. Gregory T. Wolf: Consulting or Advisory Role—Merck; Regeneron; Brooklyn Immunotherapeutics; Research Funding—Brooklyn Immunotherapeutics. Francis P. Worden: Consulting or Advisory Role—Bristol‐Myers Squibb; Loxo; Merck; Research Funding—Bristol‐Myers Squibb; Eisai (Inst); Loxo; Merck (Inst); orgenics; Pfizer (Inst). The remaining authors declare no conflicts of interest.

## Supporting information


**Figure S1:** 2 × 2 KM plots.
**Figure S2:** 2 × 2 Scatterplots (correlations).
**Figure S3:** 2 × 2 KM plots.

**Table S1:** Positive predictive value and associations between ratios and IC response subset analyses by disease site and Tstage. PPV, positive predictive value. *p*‐value from chi‐square test.
**Table S2:** Adjusted Cox model results.

## Data Availability

The data that support the findings of this study are available on request from the corresponding author. The data are not publicly available due to privacy or ethical restrictions.
