# Supplementary material for: Neutrophil to Lymphocyte and Lymphocyte to Monocyte Ratios Predict Improved Survival and Response to Induction Chemotherapy in Locally Advanced Squamous Cell Carcinoma of the Larynx
Source: Head Neck. 2025 Dec 16;48(5):1311–8. doi: 10.1002/hed.70132 (PMC13055421; doi:10.1002/hed.70132)

**Supplemental Figures**

**Figure S1: 2x2 KM plots**

**NLR historical cutpoints**

**LMR historical cutpoints**

**Figure S2: 2x2 Scatterplots (correlations)**

**CD4- NLR and LMR**

**CD8- NLR and LMR**

**Figure S3: 2x2 KM plots**

**LMR LFS**

**NLR LFS**

**Supplemental Online Figures**

**Supplemental Figure 1. Ratios and survival over time.** Kaplan Meier curves of overall, disease-specific survival time stratified by historical cut-points based on NLR and LMR


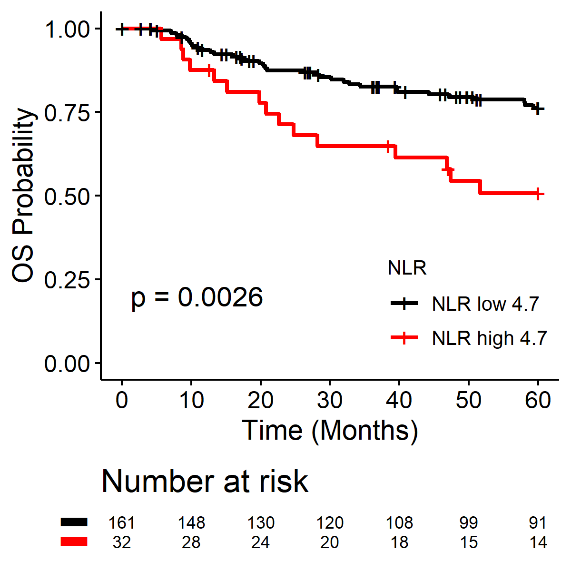

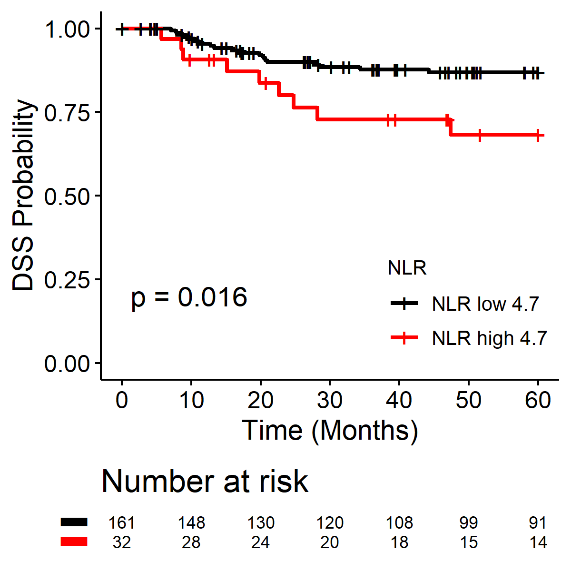


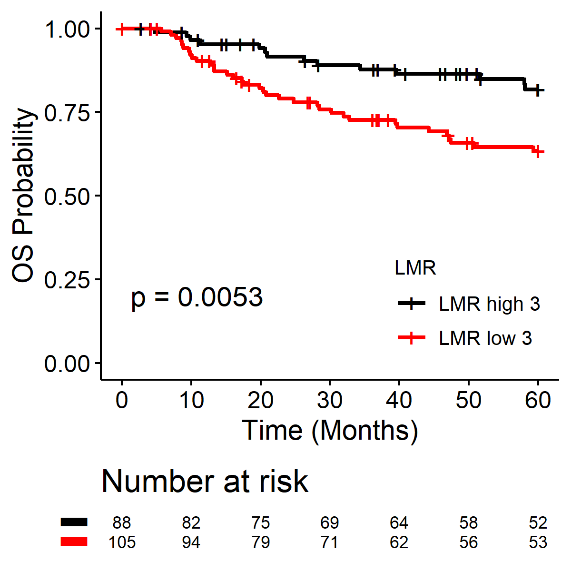

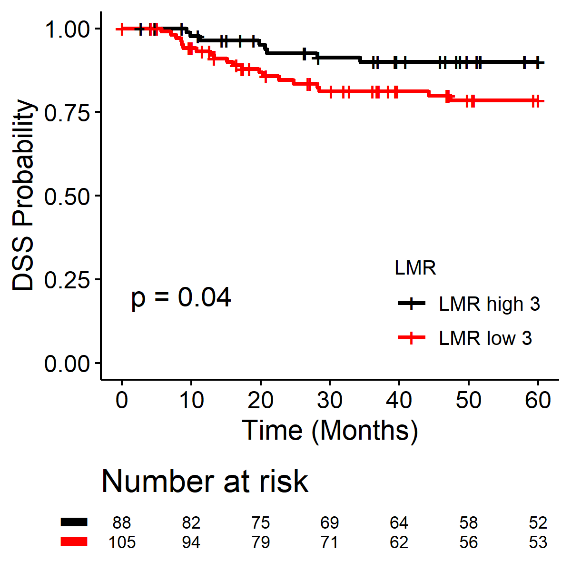


**Supplemental Figure 2. Association between absolute lymphocyte count and circulating CD4+ and CD8+ lymphocyte counts in UMCC 9520 trial participants.**


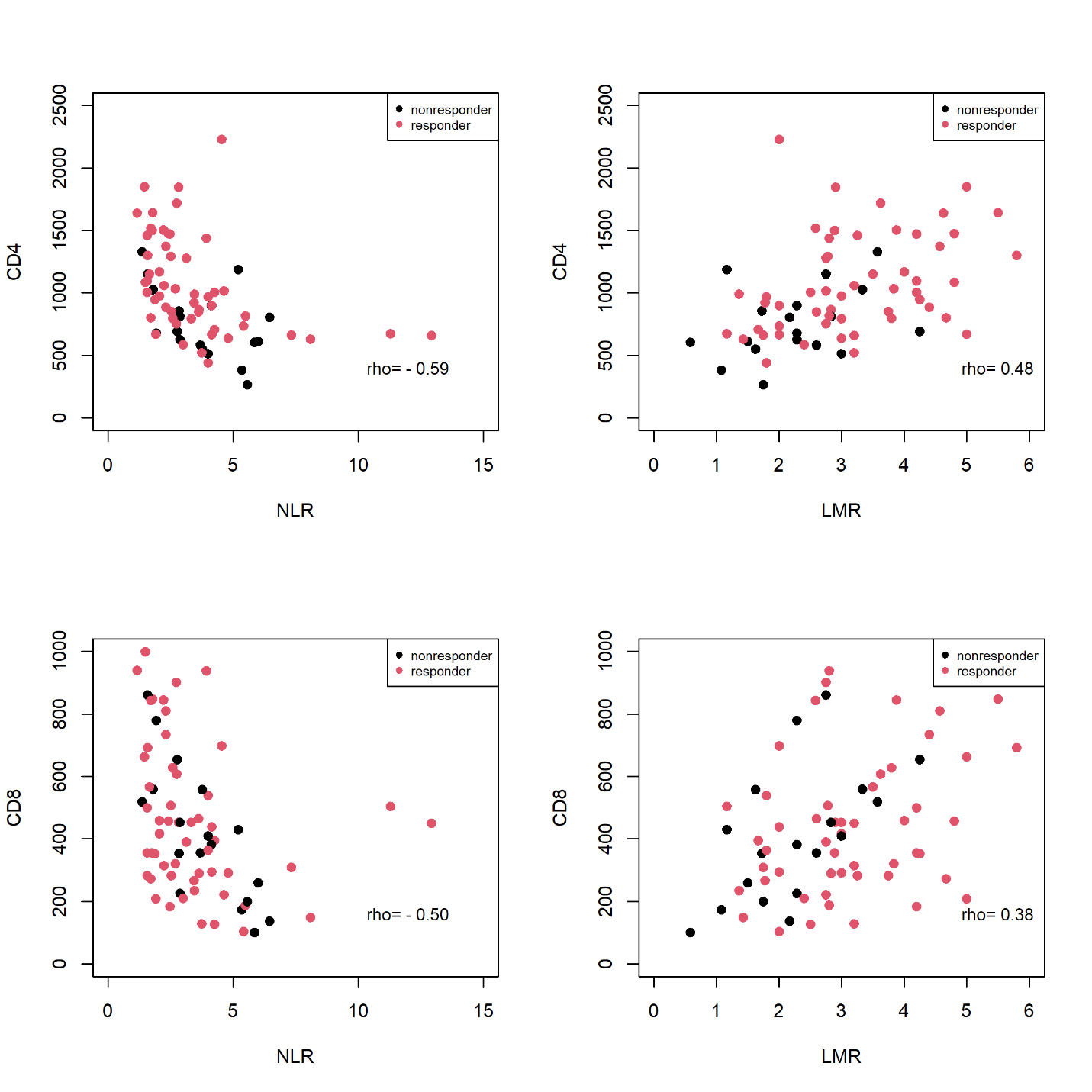


*rho=Spearman Correlation Coefficient.

**Supplemental Figure 3. Ratios and Laryngectomy Free Survival.** Kaplan Meier curves of Laryngectomy Free Survival probability prior to CRT stratified by 2.8 cut-point based on a) LMR (b) NLR.

a.


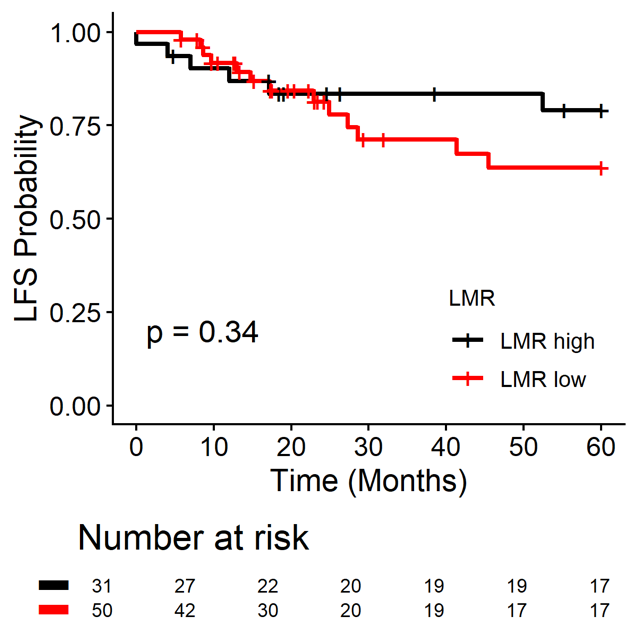


b.


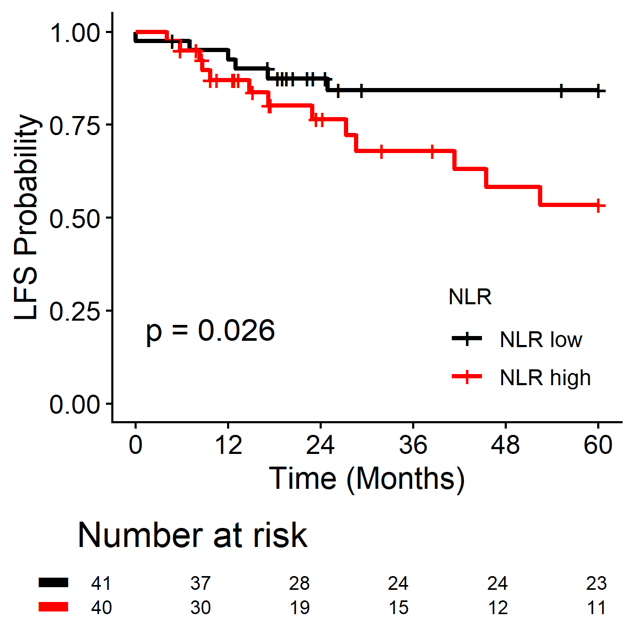

Supplement: Supplementary file 1 — Figure S1: 2 × 2 KM plots. Figure S2: 2 × 2 Scatterplots (correlations). Figure S3: 2 × 2 KM plots. [file HED-48-1311-s002.docx]
