# Supplementary material for: Neutrophil to Lymphocyte and Lymphocyte to Monocyte Ratios Predict Improved Survival and Response to Induction Chemotherapy in Locally Advanced Squamous Cell Carcinoma of the Larynx
Source: Head Neck. 2025 Dec 16;48(5):1311–8. doi: 10.1002/hed.70132 (PMC13055421; doi:10.1002/hed.70132)
Supplement: Supplementary file 2 — Table S1: Positive predictive value and associations between ratios and IC response subset analyses by disease site and Tstage. PPV, positive predictive value. p‐value from chi‐square test. Table S2: Adjusted Cox model results. [file HED-48-1311-s001.docx]

**Supplemental Online Tables**

**Table S1. Positive Predictive Value and Associations between ratios and IC Response Subset Analyses by Disease Site and Tstage.** PPV=Positive Predictive Value. P-value from chi-square test.

|  |  | Positive Predictive Value  p-value | | | | | |
| --- | --- | --- | --- | --- | --- | --- | --- |
| Ratio |  | Overall  n=193 | Glottic  n=48 | Supra-glottic  n=138 | Pyriform Sinus  n=7 | T1-T3  n=129 | T4  n=74 |
|  |  |  |  |  |  |  |  |
| NLR ≤ 2.8 | good | **83/99**  **84%**  **0.04** | 25/29  86%  0.51 | 55/67  82%  0.07 | 3/3  100%  0.35 | 55/68  81%  0.56 | **28/31**  **90%**  **0.01** |
| LMR ≥ 2.8 | good | **87/102**  **85%**  **0.0007** | 24/26  92%  0.07 | 58/71  82%  0.08 | 5/5  100%  0.09 | 58/70  83%  0.22 | **29/32**  **91%**  **0.009** |
|  |  |  |  |  |  |  |  |
| NLR or LMR | good | **102/122**  **84%**  **0.01** | 28/32  88%  0.27 | **69/85**  **81%**  **0.04** | 5/5  100%  0.09 | 68/83  82%  0.23 | **34/39**  **87%**  **0.01** |
| NLR and LMR | good | **68/79**  **86% 0.02** | 21/23  91%  0.16 | 44/53  83%  0.10 | 3/3  100%  0.35 | 45/55  82%  0.48 | **23/24**  **96%**  **0.005** |

**Table S2 Adjusted Cox model results**

|  |  |  | Overall Survival | | | Disease Specific Survival | | |
| --- | --- | --- | --- | --- | --- | --- | --- | --- |
|  |  |  | HR | 95% CI | p-value | HR | 95% CI | p-value |
| NLR good | NLR ≤ 2.8 |  | 0.6 | 0.4-1.0 | 0.05 | 0.5 | 0.3-1.1 | 0.10 |
|  |  |  |  |  |  |  |  |  |
| LMR good | **LMR ≥ 2.8** |  | **0.3** | **0.2-0.6** | **<0.0001** | **0.4** | **0.2-0.8** | **0.01** |
|  |  |  |  |  |  |  |  |  |
| Combination |  |  |  |  |  |  |  |  |
| good, good | LMR ≥ 2.8 + NLR ≤ 2.8 |  | ref |  |  | ref |  |  |
| good, poor | LMR ≥ 2.8 + NLR > 2.8 |  | 0.9 | 0.4 - 2.4 | 0.90 | 1.1 | 0.3-3.5 | 0.86 |
| poor, good | LMR < 2.8 + NLR ≤ 2.8 |  | **2.5** | **1.2-5.4** | **0.02** | 1.8 | 0.6-5.6 | 0.46 |
| poor, poor | **LMR < 2.8 + NLR > 2.8** |  | **3.1** | **1.6-5.7** | **0.0004** | **2.9** | **1.2-6.8** | **0.01** |

*adjusted by controlling for ALC in Cox model.
